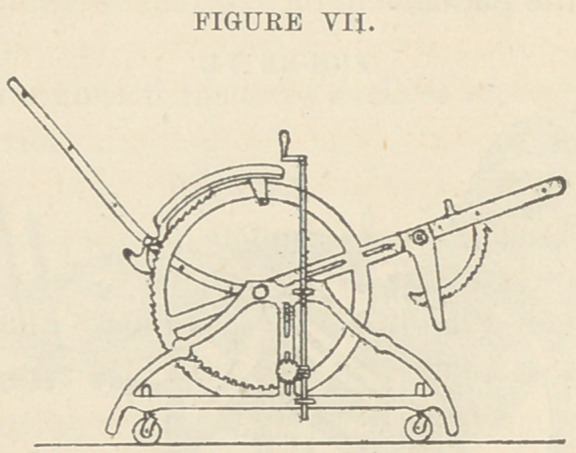# A New Resting and Invalid Chair

**Published:** 1877-09

**Authors:** Ephraim Cutter

**Affiliations:** Cambridge, Mass.


### EXAMINER.

Vol. XXXV.—SEPTEMBER, 1877.—No. 3.

### A NEW RESTING AND INVALID CHAIR.

Ephraim Cutter, M. D., Cambridge, Mass.

The philosophy of rest in chairs, embraces some of the following principles :

1st. The weight of the body necessitates rest upon some support during its physical existence. In the standing position, the feet must be supported upon fixed or floating material.

It is proposed to confine these remarks to supine, horizontal, or inclined positions.

In each case the body is in a state of perfect rest, when it is supported at as many points of contact as possible between it and the supporting material. The weight must be distributed as evenly as possible over the largest extent of surface, for then there is a minimum of compression of the tissues. When a swimmer is floating in water, the surrounding fluid is applied over the body, and the weight evenly distributed over the largest amount of surface. But water cannot be employed as a support except in a bed, since its chemical and physical qualities prevent its adoption for a chair except in the condition of ice.

Dry material used for a chair, to be thoroughly adapted as a support, must be accurately fitted to the whole posterior surface of the body. It should be a non-conductor of heat; elastic or non-elastic ; ventilated or not ventilated.

2d. The position of the human body at rest should be such as to allow the least amount ot expenditure ot energy, in order to conduct the circulation of the blood. If there is local compression of any part, there the circulation is impeded ; the resistance to be overcome requires the heart to put forth more force, or nutrition becomes impaired. When the pressure is long continued in the case of sickness, bed-sore often results. The posture in which the circulation is the most easily performed, is that in which the thighs are flexed and the feet supported higher than the head. This position avoids hydrostatic pressure of the blood in the veins of the lower extremities, amounting sometimes in tall persons standing, to several pounds to the square inch, the numerous valves in the same veins also obstruct the passage of the blood. Now in the case supposed, the blood gravitates towards the heart, and facilitates its own return. A quickened circulation means improved nutrition, hence, surgeons used formerly to elevate the lower limbs in the treatment of ulcers of the leg. It means also comfort, hence, the habit acquired by our legal brethren of putting their feet upon chairs and mantels. The habit of sitting in chairs tipped backward is a partial attempt at gaining rest by the same process, a habit which has been regarded by foreigners as a national reproach to us.

But it should not be dismissed with a sneer, for it is a partial carrying out of a principle which faciliates *rest*; because, by it the blood is more readily returned through the veins, aided by it own weight plus the absence of hydrostatic pressure ; and because there is a saving of heart-force. A few moments of heart-rest are not only refreshing but necessary.

This may not amount to so much in health as in disease. Take for example, a case of valvular disease of the heart; there the organ works on under great disadvantages, it is like a pump with one box deranged. It labors hard to push forward the life stream. Sometimes it fails suddenly and the result is death ; but generally it succeeds in maintaining the circulation. This is done, however, at the expenditure of a great amount of vital force. If the other organs of the body, are sound and carefully managed, this strain is readily borne, but if the body becomes from any cause wearied, the strain is felt, and palpitation and labored respiration result.

Now if in such a case, the lower extremities be elevated, so that the body will represent an angle somewhat larger than that of the letter V; the head being at the left extremity of the V, the hips at the angle, and the feet at the right hand extremity, then we have the position in which the blood gravitates towards the centre of the body. The heart has the minimum of work to perform, and the conditions of good physical rest are attained, because the body touches its posterior planes, curves and surfaces from head to foot.

A man, aged about 61 years, furnished a practical illustration of the effect of this position. He had organic disease of the heart, indicated by palpitation, only one cardiac sound, dyspnœa, sleeplessness and inability to lie in bed over two hours at a time in the night. He had overworked in mind and body, and thus aggravated all his bad symptoms. At the writer's request he assumed the V position on a chair [to be described], and after two days use of it, his heart became so much rested that he found no difficulty in sleeping all night in bed. Without employing medicine, and aided by rest alone, his heart, relieved of its overload, regained power enough to do its work under the unfavorable conditions of valvular lesion.

3d. Paradoxical as it may seem, rest for the living human body requires motion. The body is a bundle of kinetic energies. It is unlike inanimate bodies in this, that if kept in one position, the joints become stiff, the body cramped; and, if one single position, no matter how comfortable, be long maintained, the person becomes incapable of normal motion. Let a strong, healthy man lie in bed continuously for weeks,—as in fractures of the thigh,—and he will become incapable of rising and walking at one effort.

It is then desirable, after having arranged for a perfect contact of the under surface of the body, to have the medium of support capable of motion at the great natural points of motion; viz., the hip and the knee.

*a* back. *b* thigh *c* leg *d*

Let *a*, *b*, *c*, *d*, represent the support in question. At *b*, place a joint moving in a vertical plane for the hip. At *c*, place a like joint for the knee. It is seen at once that a variety of movements may be executed with this device, analagous to those of the body, as follows:

Elevate *a*, *b*, to a right angle with the *b*, *c*, *d*.

Suppose a man extended upon this support, and we have the very comfortable chair found in the Boston barber-shops. Here the weight of the body comes upon the legs and thighs. The hydrostatic pressure is lessened by the height of the lower limb, and the heart column is correspondingly relieved.

But if long maintained, this position becomes irksome. A healthy person can change it, but the invalid cannot. How, then, can we give this support, and permit a change?

Simply by putting the support on a frame work and jointing it at *b*, so that the *V* rotates 90°, upward.

FIGURE I.

*a*, back; *b*, thigh; *c*, leg.

Fig. I. represents this motion; *a*, *b*, *d*, is the position described; *a'*, *b*, *d'*, the same when the support is turned back. The angle *d'*, *b*, *d*, represents the angle of inclination of the thighs. The dotted line *d'*, *d*, represents the hydrostatic pressure relieved; *a''*, *b*, *d''*, the *V* position exaggerated, as *d''* is higher than *a''*.

When *a''* is of the same height as *d''*, the weight is divided according to the weight of the parts.

*A'''*, *b*, *d'''* represents the whole weight of the body thrown upon the back,—a theoretical, but not a practical position, the feet being elevated. Both body and legs are here reversed 90° from the position *a*, *b*, *d*.

Here, then, support and motion are combined so as to throw at will the body-weight on the back or thigh, or to divide it equally between both, the division being in any proportion desired. This arrangement is advantageous, because it distributes the pressure evenly, and yet allows of change. Still, it involves immobility of the great joints. By referring to the diagram it will be seen that it is easy to get more motions by using the joints in the support *a*, *b*, *c*, *d*. In Fig. 1, bend down *c*, *d* to a vertical position, and *a*, *b*, *c*, *d*, will represent a common chair position. Rotate this backward 45° upon the supposed frame and the *W* position is attained when the thighs and legs are flexed, and yet perfect support attained.

This forms the "triple inclined plane" of Dr. Lewis A. Sayre, of New York city, with this exception, that the joints in the described device are movable and adjustable. It is an excellent, theoretical, and practical position for persons suffering from fracture of the thigh. The weight of the body gives the extension, the position of the legs, the counter-extension. Being fitted to the sound thigh, of course the broken thigh is constantly drawn into place. The joints are, or can be, kept moving while the extension is continued. As motion is the natural condition of a joint, of course any device which allows of proper extension with absolute and relative motion (change) is a desideratum.

FIGURE II.

*a*, back; *b*, thigh; *c*, leg.

Fig. II. represents a change in the position first given, so that the head approaches the floor,—a position which is useful in case of fainting from any cause.

With this device *a*, *b*, *c*, *d*, hung at *b*, on a frame, any position of the human body, sitting or reclining on the back, may be imitated. More than bed or lounge, or any previously offered device, it offers the opportunity of depressing the head and trunk almost to the floor.

With these preliminary observations, the writer desires to call attention to the contribution he offers, in solution of the mechanical problems proposed.

FIGURE III.

Fig. III. Mechanical drawing of author's chair.

The frame is made of cast iron, heavy enough for durability and safety. The back is curved to fit the body and varies in length from 24 to 36 inches. The sides are formed by circles, in order to get the strength of the double arch. Notches are cut on the posterior and upper quarters of the circles, in which play pawls attached to and regulating the back portion. Teeth are cut on the inside of the lower and posterior quarter, in which play cogs that are turned by an endless screw on a shaft attached to the side of the frame-work. This screw ensures control of the apparatus and regulates its motion. It may be turned by the patient. The circles, also, afford support for the arms of the chair. An iron cross-bar traverses the horizontal diameter of the circles. This affords strength, and extending forward, forms an attachment for the seat portion and the bracket for the leg portion. It is fenestrated so that the seat portion can be lengthened. The leg portion consists of a U attached by a joint to the end of the seat portion. Half way down on each side are long pawls with teeth cut on the convex side which engage in the brackets of the seat portion.

Suitable horns project in front from these pawls to permit their being lifted when it is desired to flex the legs. To extend the leg portion it is only necessary to draw it out upwards.

*Foot Rest.* This was attached to the earlier chairs, but has been abandoned as, unless fitted accurately to every leg, it prevented the accurate resting of both thigh and leg upon their support. Besides, the device is intended to relieve the feet from pressure. When a chair is intended for the use of only *one* person a foot rest may be applied.

The thigh portion measures 17–22 inches. The leg 17 inches. The width of the chair varies from 18 to 25 inches.

Three inch castors are placed under each corner of the frame. The size of the frame was determined by actual test, and the length of side was governed by the stability under ordinary circumstances. Invalidism must have a chair that will not easily tip over. Remove the castors and the chair becomes almost a fixture.

FIGURE IV.

Bed position of the chair.

FEATURES OF THIS CHAIR.

(*a*). Center of support, the center of motion.

(*b*). Motions, those of the hip and knee joints, in vertical planes.

(*c*). Absolute motion without relative motion of body, thigh and leg.

(*d*). The reverse of the last.

(*e*). Accommodation to abnormal conditions, fractures and anchylosis.

(*f*). Person can change the part rested upon. In bedridden cases, this is of service in preventing bed-sores.

(*g*). A person can be raised from a supine to a sitting posture (or the reverse) gradually, and stop at thirty-four points on the way.

(*h*). Adjustments to different lengths of thighs.

(*i*). Patient may be removed from bed to chair without lowering.

(*j*). Absolute motion made by endless screw and pawls of the simplest forms.

USES.

1. *For orthopnœa*, as in asthma, heart disease, rheumatism, and where persons are obliged to sit up all night and cannot lie in bed.

2. *For Fracture Bed.* See sequel.

3. *For Operating Chair*, this was the original design of the chair to be used in modified thyrotomy as published by J. Campbell & Son, Boston, 1867.

FIGURE V.

Fig. V. Device for holding a child in the operation upon the thyroid cartilage.

For surgical operations of other kinds, it permits close contact of the operator and fixation of patient. Faintness from hemorrhage is controlled; vomiting is readily managed by elevating to a vertical position.

As a *gynecological* and *office* chair, I have found it useful. See sequel.

(4.) In convalescence and invalidism it is a useful means of getting a patient out of bed so as to use an ordinary chair. See sequel.

(5.) *In health*, it may be used as a reclining and resting chair. Women generally find it very restful in backache.

They are sold from $35 to $45; are durable, and will last for a generation with ordinary care. The upholstering varies with the tastes of the patient.

FIGURE VI.

Chair with Fracture apparatus, which may be detached by removing one screw.

The device of the author represented in the cut, is for extension in fractures of the thighs or legs. It is held securely in place by one screw. Extension may be made by a weight attached to a cord that passes over a pulley, or by an india-rubber tube fastened to the slot in which the pulley plays. By this arrangement the joints are kept in motion without disarranging the extension. The patients may sit up for the sake of eating, reading or writing. For defecation, &c., a bed pan is provided, made somewhat like a common dusting pan. The patient may be moved about the apartment, up to the window or even into another room. In this manner the tedium of confinement is very much alleviated. This relief is by no means an unimportant element in securing a successful and satisfactory progress of the case. The elevation of the head and the motion of the great joints ensure a shorter convalescence.

The shocks, also, of motion and jars of the apartment, ordinarily so troublesome in rheumatic fever, are not felt, as the suspension of the patient is all upon one centre.

The following cases of fracture have been treated in the manner described :

FIGURE VII.

Case I. V position : a middle-aged, medium-sized, robust man, aged 50 years, was thrown from his carriage early in July, 1872. He sustained a compound fracture of the right ankle joint, the lower portion of the thigh presented a Y fracture, and the malleolar process protruded one and one-half ($1\frac{1}{2}$) inches through the skin. The ankle joint was laid open. He was placed upon the chair in the V position, the thigh and seat portion being on an elevated single plane. A fracture box was placed on the lower part of the chair, and the projecting part of the box supported by a stand. The fractured limb was surrounded with sponge soaked in glycerine and packed in three pads of linen, one below and one on either side of the leg ; the pads were covered with oil-cloth near the side of the wound, no bandages or circular compression of the limb were employed.

At night, the back portion of the chair was lowered to the level of the other part ; in the day time, it was raised to any desired elevation. He had during the course of treatment, pneumonia, secondary fever from inflammation and purulent infiltration of the joints and surrounding parts. The wound discharged at least one pint of pus. When he stood on his feet for the first time he *immediately* walked through the hall to an adjoining room, an exceptional circumstances in my experience of similar fractures, the reason being that an enforced recumbent posture for seven weeks or more, causes an accommodation of the system's circulation adapted to the posture. Any sudden change from the dorsal to the erect position is then followed by a disturbance of the nice economy of the heart-force, so that faintness results. The arrangement of this patient's bed allowed of frequent changes of position, so that there was no disturbance of the central circulation, and locomotion became immediately possible. This patient made a good recovery in joint and limb.

Under date of June, 1877, he writes :—"The relief on being placed on the chair after lying upon the bed for a few days was great. I was not taken from it for *ten* weeks, during the hottest of weather, when a bed is almost unbearable. I could eat, sleep or write upon it; my position could be changed at any time, and this afforded much relief. It (the chair) is easily managed, and, I can honestly say, it is the best and most comfortable I have seen. Having tried it, I know."

This was the first case of fracture treated in the chair, and thus it possesses an unusual interest.

Case II. A boy, eight (8) years of age, very nervous and pale, was thrown off a pony in March, 1875, and sustained a simple fracture of the right femur, middle third. Fears were expressed as to the effect of the shock upon his nervous system, in view of the fact that he had been difficult to manage whenever he had been sick before. A chair was arranged with a ten-inch thigh portion. The back of the chair was upholstered with Brussels carpeting, the seat with sole leather and the leg portion with carpet. He was transferred to the chair as follows: Chair placed in the horizontal position; a pole six feet long and two inches in diameter was held over the boy as he lay in bed. A blanket on which he lay was then drawn over the pole from both sides of the boy, gently made tense and fastened by tacks driven through the blanket into the pole. He was then raised by the ends of the pole and placed upon the chair so easily that he did not know when it was done, as he could not see. Adhesive straps were fastened to the thigh and connected with a weight simply hung over the back of the chair. This furnished the extension. To ensure safety, a sole leather splint was placed around the fractured thigh. The chair was continually moved about, and the back, thigh and leg portions were repeatedly moved backwards and forwards, giving motion to the hip and knee joint without at all disarranging the extension. A common dust-pan covered with newspaper served for a bed utensil. In a few weeks he made a perfect recovery, so much so that his uncle said he could not tell from his gait which limb was broken. The quick recovery must be attributed partly to his youth, partly to a diet of animal food and unbolted grain, and partly to the movable and adjustable bed chair.

Case III. Miss A. R., aged 70 years, single; fell while crossing the oiled hard pine floor of her kitchen in April, 1875. She had been in miserable health during the fall and winter previous, with digestive troubles which an autopsy shows to have beeen due to cancer of the liver. Her debility was so great that she kept swooning away after the fracture, and it was with great difficulty that she could be kept from dying, by the use of diffusible stimulants. She was, however, placed upon an invalid chair. Adhesive plasters, an India rubber tubing, and a fixed point beyond the foot furnished the means of extension. The tubing furnished an adjustable, direct, elastic extension in a small compass. At the very outset of her confinement in the chair, Miss R. fainted away frequently from sheer weakness. Whenever this occurred, it was only necessary to turn the chair backward and to lower the head until the equilibrium was restored. At the time it seemed as if this change of position alone saved her life, as the fainting fits were so long continued that medicines lost their effect, the gravity proving more valuable than the drugs. A singular and unusual experience followed this, in that she had an attack of diphtheria, the membrane being abundant and visible in the throat. There was fever, and cervical adenopathy. At times she was choked by the detached membrane, and when this occurred, she was rapidly brought to an erect position. She was thus enabled to manage her throat and mouth, as she could not have done in the ordinary horizontal position. So that in fainting and vomiting she tested the chair quite thoroughly. Curiously enough, the fractured bone united well and promptly with three-fourths of an inch shortening. When it was healed she was replaced in bed.

Her miserable and painful existence, however, was in a few weeks brought to a conclusion by cancer of the liver. The organ was found to be enlarged, and studded with yellowish globules of considerable consistency. Her life certainly seemed to have been prolonged by the use of the chair.

### Case IV.

In Dec., 1876, Mr. J. D—— tried to remove some ice from his boot-heel by kicking, and fell. When raised he was found to have sustained a dislocation of the left ankle joint, and a complete fracture of both malleoli. The dislocation was relieved by bystanders. He was placed upon a cane chair covered with a hair mattrass. The extension apparatus represented in the cut, was employed in this case. It worked well. There was no difficulty in assuming comfortable positions. The malleoli became rapidly attached in place, but the ligaments and torn fascia were as usual, long in healing. The patient and his friends expressed great satisfaction with the use of the chair, as it relieved the tedium of confinement. Recovery was good, though the patient was a man of full habit, very strong and muscular, and unused to being shut up in the house.

### Case V.

Dr. S. W. Kelly, of North Cambridge, Mass., aged 55 years, was walking down Hanover street, Boston, when he suddenly found himself lying upon the icy pavement. He had slipped over an iron coal scuttle. On examination he was found to have a dislocation of the ankle and fracture of the internal malleolus of the tibia.

His subsequent experience was almost the same as that of Case IV, though being of a nervous temperament the confinement was more irksome. He, also, had a slight attack of inflammation of the lungs. He made a speedy and good recovery, and says that he can conscientiously recommend the chair from personal knowledge. He is a regularly educated physician and a member of the Massachusetts Medical Society, in good standing. He has had a large experience in the practice of his profession.

CASE VI.

Mrs. A. W., of Boston, while visiting in Dec. last, fell and sustained an impacted fracture of the neck of the femur. She was a widow 82 years of age, and of active habits. The diagnosis was concurred in by three surgeons. She was not rendered helpless. With assistance she could change from bed to lounge or chair. There was inability to use or move actively the injured limb. For the first month she used a common Holmes' reclining chair. She was, however, not satisfied with it, and later made trial of the device herein described. This proved so satisfactory that she declared she gained "two days in one" by its use. No extension or bandage was used, the position on the chair being solely relied upon. She has made a remarkable recovery considering her age, with but one-half inch shortening. She walks up and down stairs, and about the house with a cane, and now has gone to the sea-shore for the summer. To the writer, this case has shed a new light upon the treatment of fractures of the thigh. Is it not a mistake to invariably apply bandages and extension? The physical changes resulting from the injury must obstruct the circulation in the limb. This may be called a *central* obstruction. Is it well to add a peripheral obstruction by bandaging? This patient might have recovered by the aid of other appliances, but it is not disparaging them to say that the chair last used certainly did well and facilitated recovery.

3. As an *operating chair*. Dr. Gilman Kimball, of Lowell, has employed it in a case of amputation of the breast. He expressed himself as pleased with it. In ordinary chairs, the patients when anæsthetized slip downwards. With this device this difficulty is obviated by simply using the chair as a V and elevating the feet. A point of resistance is thus formed. In a late case of modified thyrotomy for the removal of a sessile growth that lined the whole larynx, the writer found his chair very useful. The operation was difficult, and protracted for one and a half hours. The patient was put (just where she was wanted) into positions impracticable by any other means known to the writer. The facility with which the operator placed himself in a suitable relation with the patient, relieved the arduousness of the trying dissection.

As a *gynecological chair*. The writer has placed women upon their backs and sides in the chair, and then depressed their heads, so that the sun's rays shone directly on to the os uteri through the speculum. Direct or reflected artificial light may be used for the same purpose, as follows: Drop the leg portion to a right angle with the thigh portion, the back being slightly elevated. The patient sits down on the seat, then lies down in the dorsal or lateral position. A few turns of the endless screw depresses the head and elevates the buttocks so that the operator, sitting or standing, can look downwards just as in writing or eating, *i. e.*, the most natural position for observation. I have used the chair in exploring the uterus by Simon's method—that is, by passing the whole hand and half the forearm into the rectum and large intestines. The peculiar position given by the chair was found to be very favorable.

I have also used the chair in measuring and fitting a pessary. The following statement indicates what the patient herself can do with the chair. A lady suffered much from an ulcerated and hyperæsthetic condition of the uterine cavity, which intrauterine topical applications alone benefited. Circumstances of time and distance forbade the frequent attendance of the writer, as often as was necessary. The patient asked, if she could not be instructed to make applications herself. The attempt was made with perfect success. Lying on the chair on the back, opposite a window, she applied the Storer speculum, held a mirror between her knees, saw the os in the mirror (this I verified,) and applied her medicament with a Pinkham's scarificator. This instrument, like a uterine sound, is grooved longitudinally, the medicament is rubbed up with lard and deposited in the groove near the extremity. A small pledget of cotton is placed in the groove next the proximal end. A wire traverses the groove, and one end of it rests on the cotton, the other projecting. When the instrument is introduced into the uterus to the point desired, it is only necessary to push forward the wire. This advances the cotton pledget, and extends the ointment.

The same patient has applied slippery elm tents, in a similar manner through the speculum.

In *convalescence.* Sometimes patients exhibit a determined inclination not to sit up in an ordinary chair, after a long sickness, when it seems as if they might do so. An instance of this occurred to the writer. The patient was induced to use the chair. At first the bed position was used. The erect position for the trunk was gradually assumed, then the legs were dropped. The spell was broken; the invalid went down stairs to dinner after two days use of the chair.

Another lady, suffering with fibroid tumor, found the chair a great relief to her pain and weakness.

Indeed it is useless to multiply the histories of such cases. As a resting chair for over-worked persons, it has produced practically the happiest results.

The chair is not a proper locomotive chair. It does not answer every imaginable purpose, but in the instances that have been named, the expectations raised were, humanly speaking, fully realized.

## Figures and Tables

**Figure f1:**
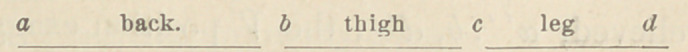


**Figure f2:**
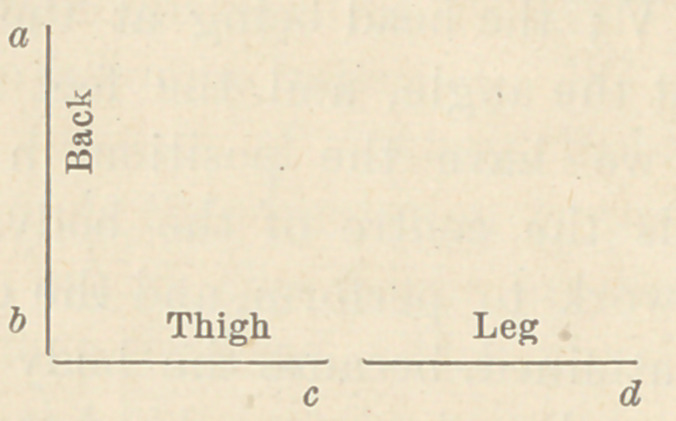


**FIGURE I. f3:**
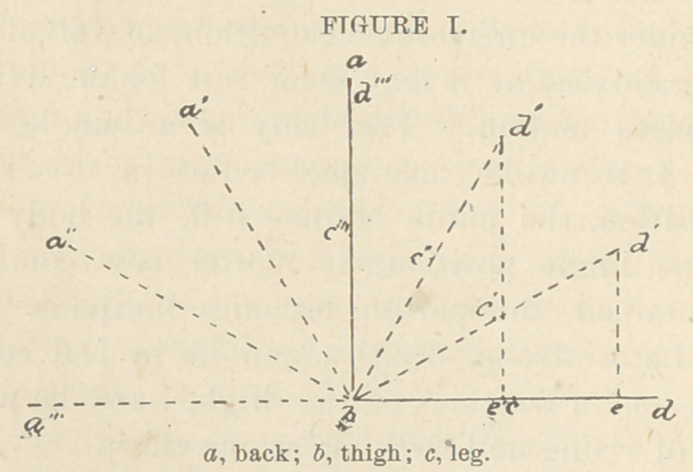


**FIGURE II. f4:**
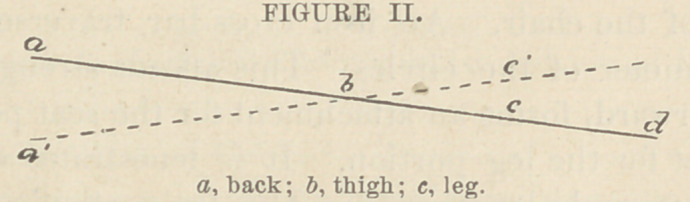


**FIGURE III. f5:**
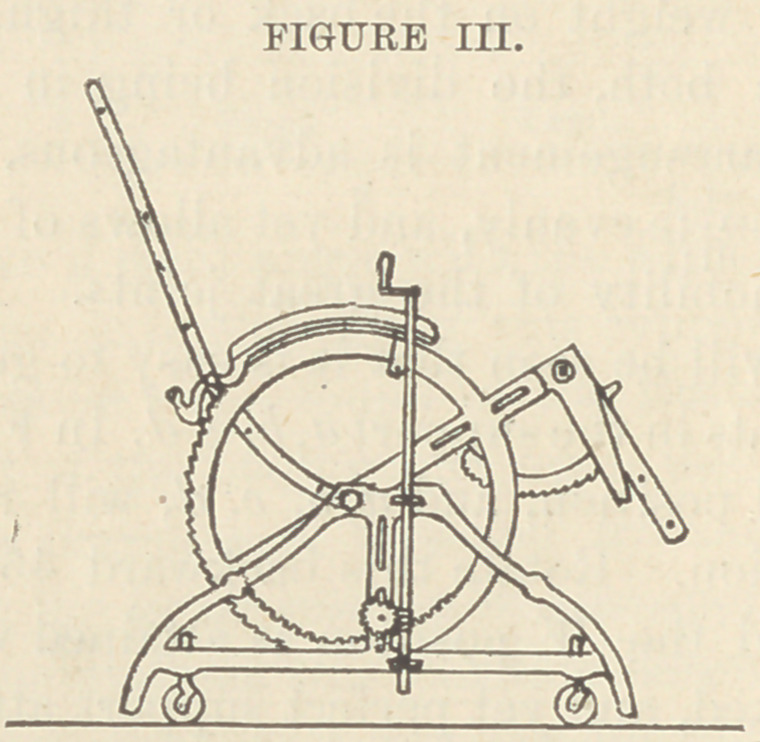


**FIGURE IV. f6:**
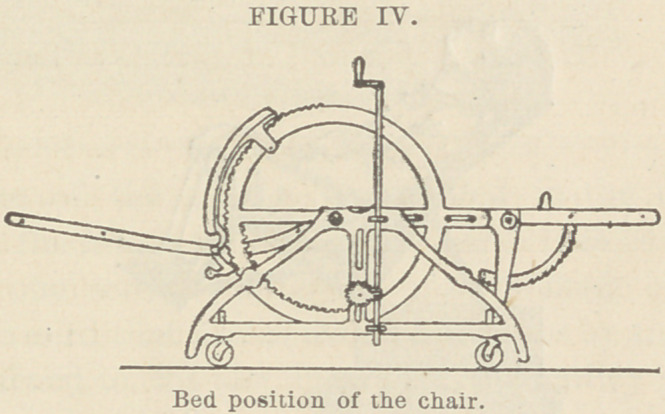


**FIGURE V. f7:**
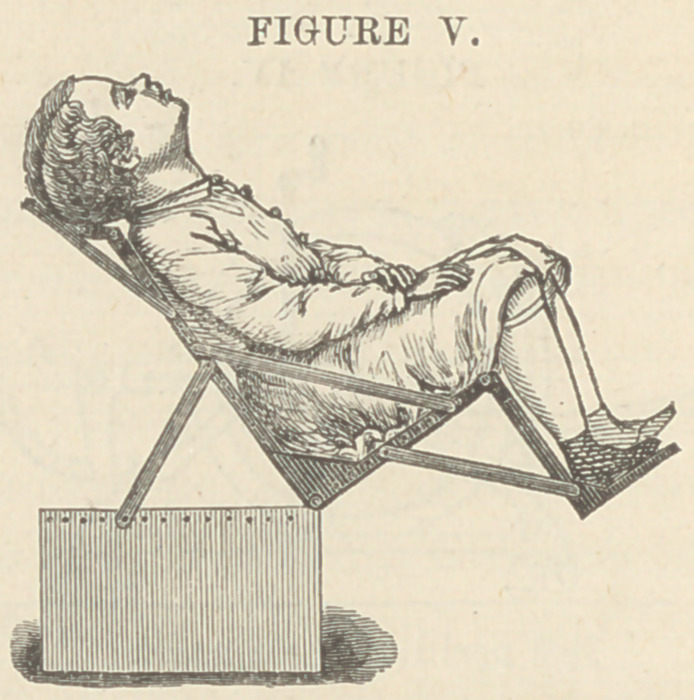


**FIGURE VI. f8:**
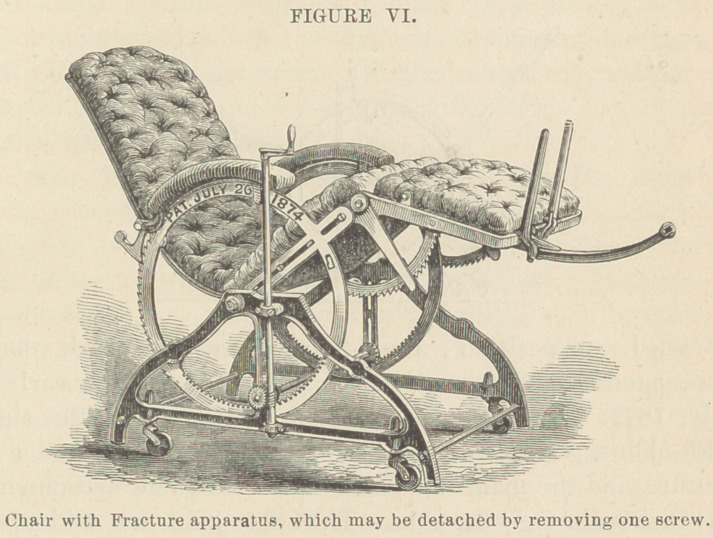


**FIGURE VII. f9:**